# PIMA-CT: Physical Model-Aware Cyclic Simulation and Denoising for Ultra-Low-Dose CT Restoration

**DOI:** 10.3389/fradi.2022.904601

**Published:** 2022-05-25

**Authors:** Peng Liu, Linsong Xu, Garrett Fullerton, Yao Xiao, James-Bond Nguyen, Zhongyu Li, Izabella Barreto, Catherine Olguin, Ruogu Fang

**Affiliations:** ^1^J. Crayton Pruitt Family Department of Biomedical Engineering, University of Florida, Gainesville, FL, United States; ^2^School of Software Engineering, Xi'an Jiaotong University, Xi'an, China; ^3^Department of Radiation Physics, University of Texas MD Anderson Cancer Center, Houston, TX, United States; ^4^Department of Radiology, University of Florida, Gainesville, FL, United States; ^5^Center for Cognitive Aging and Memory, McKnight Brain Institute, University of Florida, Gainesville, FL, United States; ^6^Department of Electrical and Computer Engineering, Herbert Wertheim College of Engineering, University of Florida, Gainesville, FL, United States

**Keywords:** physical model, medical image denoising, low-dose CT, noise removal, dose reduction, deep learning, generative adversarial network (GAN)

## Abstract

A body of studies has proposed to obtain high-quality images from low-dose and noisy Computed Tomography (CT) scans for radiation reduction. However, these studies are designed for population-level data without considering the variation in CT devices and individuals, limiting the current approaches' performance, especially for ultra-low-dose CT imaging. Here, we proposed PIMA-CT, a physical anthropomorphic phantom model integrating an unsupervised learning framework, using a novel deep learning technique called Cyclic Simulation and Denoising (CSD), to address these limitations. We first acquired paired low-dose and standard-dose CT scans of the phantom and then developed two generative neural networks: noise simulator and denoiser. The simulator extracts real low-dose noise and tissue features from two separate image spaces (e.g., low-dose phantom model scans and standard-dose patient scans) into a unified feature space. Meanwhile, the denoiser provides feedback to the simulator on the quality of the generated noise. In this way, the simulator and denoiser cyclically interact to optimize network learning and ease the denoiser to simultaneously remove noise and restore tissue features. We thoroughly evaluate our method for removing both real low-dose noise and Gaussian simulated low-dose noise. The results show that CSD outperforms one of the state-of-the-art denoising algorithms without using any labeled data (actual patients' low-dose CT scans) nor simulated low-dose CT scans. This study may shed light on incorporating physical models in medical imaging, especially for ultra-low level dose CT scans restoration.

## 1. Introduction

The quality of medical imaging is critical for diagnosis and treatments. However, medical imaging often suffers from the noise produced at either the image reconstruction or post-imaging stages. Medical physicists in radiology play several essential roles in maintaining imaging quality and stability for imaging machines, such as computed tomography (CT). They usually adopt an anthropomorphic physical model to facilitate the assessment of imaging quality and the adjustment of the imaging machines' parameters before performing on real patients. Motivated by this, we hypothesized that a physical model could also help restore high-quality images for the cases in the post-imaging stage, such as radiation reduction in CT imaging.

**Figure 1:**
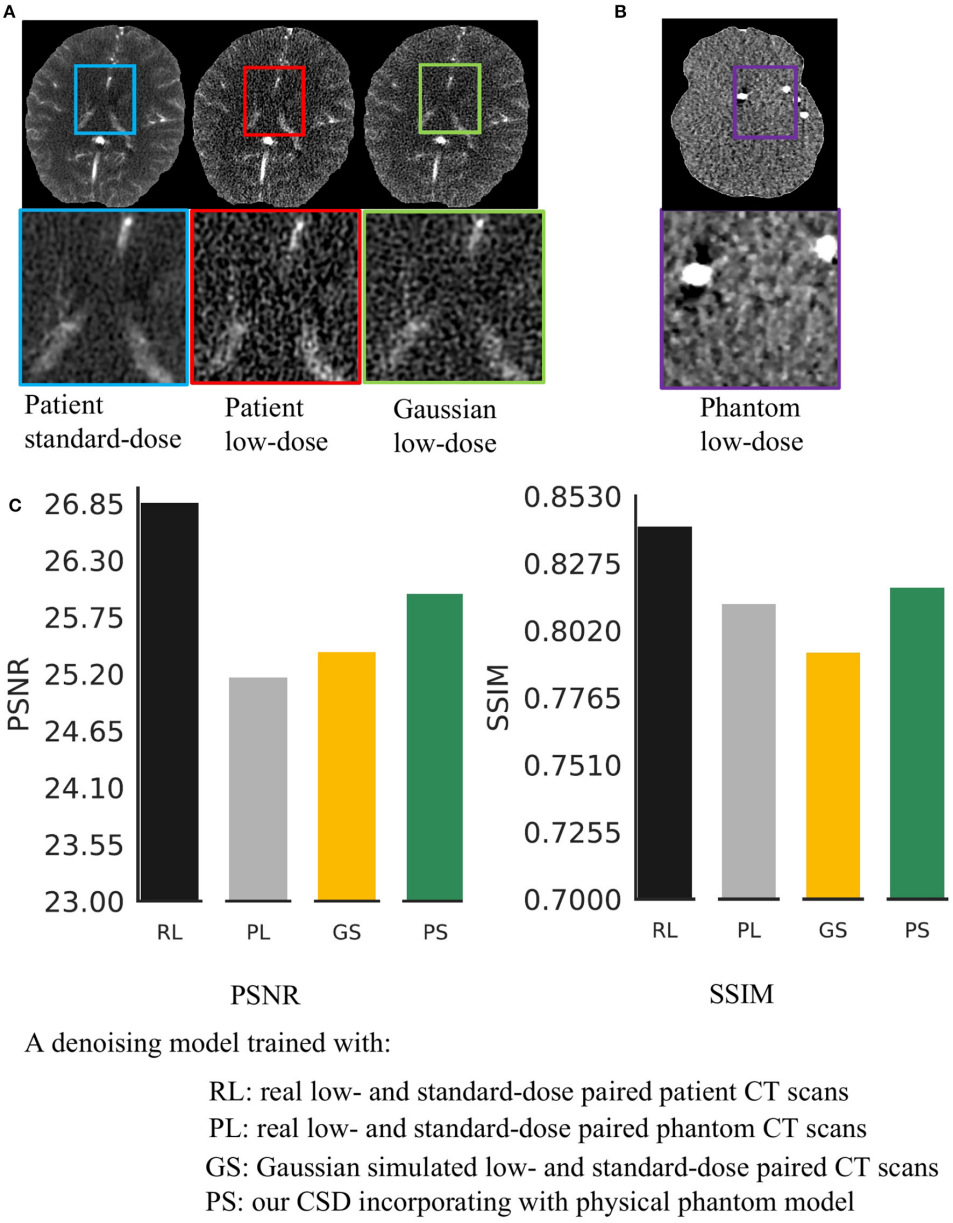
Real low-dose has a different noise distribution from Gaussian noise and is hard to remove. **(A)** It shows a visual comparison of the standard-dose computed tomography (CT), real low-dose CT, and Gaussian simulated low-dose CT scans. **(B)** It shows a low-dose CT scanned by using a physical phantom model. **(C)** We trained four same structural Deep Neural Network (DNNs) using various types of low-dose noise with the same noise level (20 mAs radiation dose) and then compared the effectiveness of noise removal.

Reducing radiation dose during imaging is a low-cost approach to release concerns about causing cancer or other negative health conditions using CT scanning (1), but this method introduces noise into CT scans, hindering the diagnostic effectiveness of such scans. Several studies (2, 3) have been proposed to address this problem by removing the noise from low-dose CT scanned images. However, these studies are designed based on Gaussian noise simulation for populations without considering the variation in CT devices and individuals, limiting the current approaches' performance, especially for ultra-low-dose CT imaging (see Figure 1A).

Moreover, most of the success of deep learning-based approaches for low-dose CT image restoration (4, 5) much relies on a large number of labeled images. However, obtaining the real low-dose CT scans is not available in practice. Accessing real image noise is critical for the development of any practical imaging algorithm. Also, real noise properties significantly vary among different CT machines and individuals. Thus, the Gaussian noise assumption is not always guaranteed in practical scenarios and significantly limits the existing approaches for ultra-low-dose CT imaging.

We address these problems by incorporating an anthropomorphic physical phantom model into generative adversarial networks. The proposed framework is named cyclic simulation and denoising (CSD). The physical model provides paired low-dose and standard-dose phantom CT scans before scanning the actual patients. These phantom scans can offer statistical noise prior, which is related to the specific CT machine for patient diagnosis, for CSD to precisely capture noise properties and remove real complex noise from CT scans. Our CSD is composed of noise simulation and denoising two networks. The simulation network facilitates the denoising network to learn real noise properties. The denoising network thus can access realistic noise through physical phantom CT scans. However, phantom scans lack tissue features (see Figure 1B). The missing tissue information prevents feasible phantom-based solutions for CT image restoration. As one can see in Figure 1C, the model trained with paired low-dose and standard-dose phantom scans fail to remove real noise from low-dose patient scans. To overcome this problem, we train CSD using normal-dose and phantom CT scans simultaneously to embrace realistic noise and tissue features into a unified learning framework without the access to labeled or Gaussian noise simulated data.

We evaluate our CSD for removing both real low-dose and Gaussian simulated noise. The results show that CSD outperforms one of the state-of-the-art denoising algorithms for ultra low-quality medical image restoration. Our main contributions include that (1) we incorporate an anthropomorphic physical phantom model into generative adversarial learning to address the challenges of removing real noise from ultra-low-dose CT scans for radiation reduction; (2) we develop an unsupervised framework in the combination of phantom CT scans that can outperform one of the start-of-the-art methods without using any labeled or other noise simulation data; (3) to the best of our knowledge, this is the first study to incorporate physical model into deep learning for medical imaging.

## 2. Materials and Methods

The problem of CT image denoising can be understood by *L* = *H* + *N*, where *H* is the clean, standard-dose CT image, *L* is the noisy, low-dose CT image, and *N* is additive image noise. Though an additive relationship does not completely represent the relationship between clean and noisy images, this formula provides a baseline for understanding the problem.

We utilize two deep networks in the framework. The first network *G*_*s*_ is the noise simulator and can be modeled by *L* = *G*_*s*_(*H*, α), where α is the desired simulated dose level and implicitly indicated in training data. The second network *G*_*d*_ is the denoiser that can be modeled by *H* = *G*_*d*_(*L*), where *G*_*d*_ is the network generating a clean image from a given low-dose noisy input *L*.

### 2.1. Unsupervised Learning by Incorporating Physical Model

**Figure 2:**
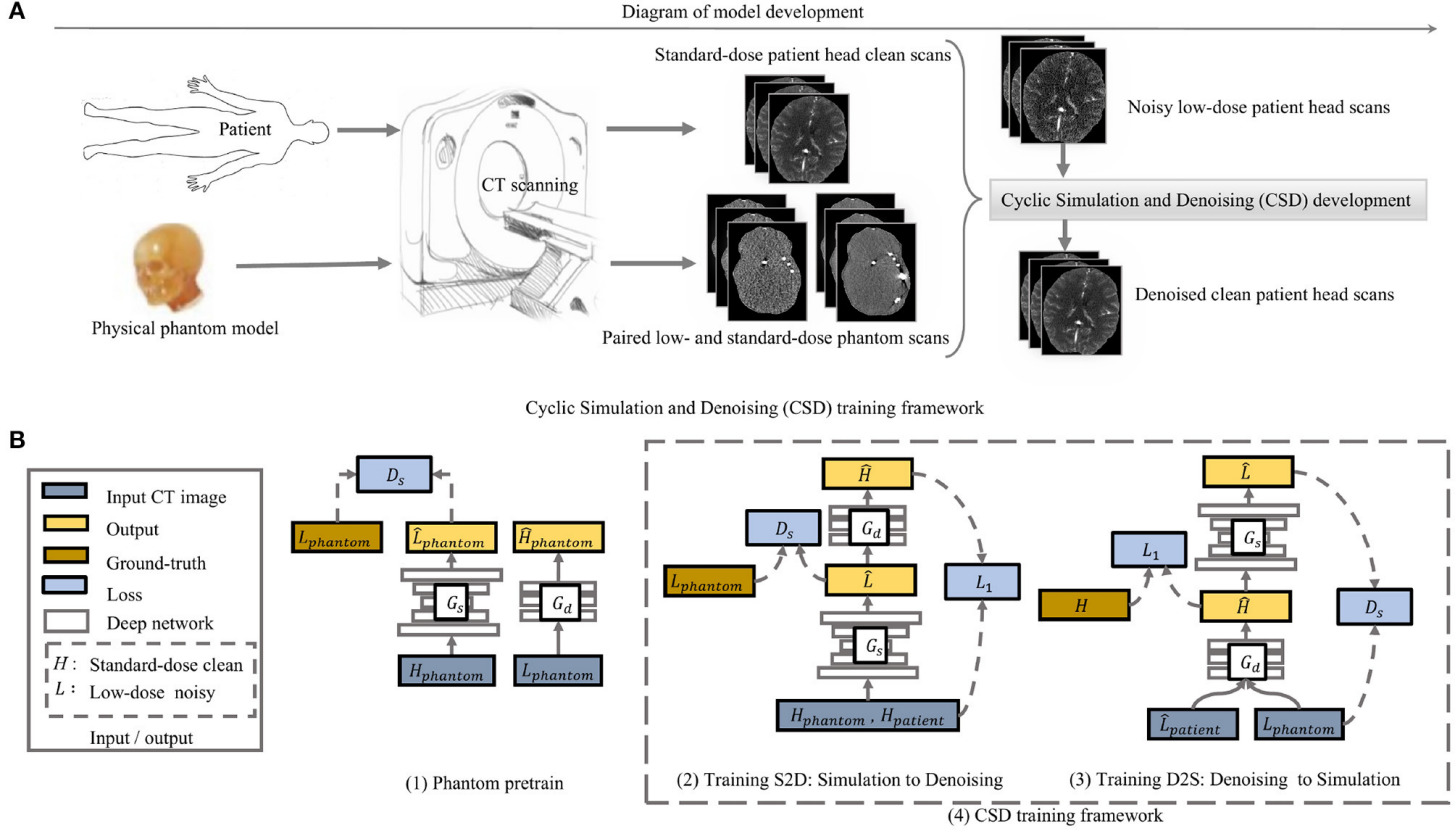
The overview of the model development in **(A)** and the proposed cyclic simulation and denoising (CSD) training framework in **(B)**. **(A)** demonstrates how we incorporate a physical phantom model into the proposed deep learning model CSD. **(B)** shows how our CSD is developed in detail. Two training stages: first, we initialize the weights of simulator and denoiser by pretraining on physical phantom CT scan (1); second, the cycle-training from noise simulation to denoising (2) and another cycle-training from denoising to simulation (3) are developed simultaneously. The *G*_*s*_ and *G*_*d*_ represent simulation and denoising, separately. During training, the two cycles interact with each other and are executed alternatively.

We use a head phantom model to obtain paired low-dose and standard-dose phantom CT scans, with which we combine the normal dose (standard-dose) patient CT scans to develop our CSD model. The phantom scans allow the model to access real noise properties and the patient scans offer the actual brain tissue features to the model. In this way, we eliminate the need for noisy low-dose CT scans from actual patients and even the Gaussian noise simulated low-dose CT scans to develop our model (Figure 2A). Therefore, we present an *unsupervised learning* framework by incorporating an anthropomorphic physical phantom model.

### 2.2. Cyclic Simulation and Denoising

#### 2.2.1. Overview

We develop two deep networks to perform simulator and denoiser individually. To ease the network training, we first use paired low-dose and standard-dose phantom CT scans to pre-train the simulator and denoiser, separately. Then, we plug the simulator and denoiser pre-trained models into our CSD framework (Figure 2A). In particular, we start with noise simulation using both the phantom and patient CT scans to generate low-dose noisy patient CT images that simultaneously provide noise and tissue features for training the denoiser (Figure 2B). Meanwhile, CSD also allows the backward training process from denoiser to simulator. The denoiser takes phantom noisy scans and simulated noisy patient scans as input to learn how to remove realistic noise and restore tissue features simultaneously, while the simulator mainly plays as a regularizer to the denoiser for stabilizing the training (Figure 2B). The interaction between simulator and denoiser forms a dynamic data-driven framework, named cyclic simulation and denoising (CSD), to address the challenges of low-dose CT image restoration.

#### 2.2.2. Pretrain Simulator and Denoiser ($H\to \hat{L},L\to \hat{H}$)

We train the simulator with a u-shape encoder-decoder generative adversarial network by formulating the objective as adversarial learning. We use a discriminator *D*_*s*_ to differentiate real low-dose CT images from fake samples generated by the simulator *G*_*s*_. We illustrate the formulation of the simulation as below.


(1) $$\begin{array}{l} L_{GAN}\left(G_{s},D_{s}\right)={𝔼}_{L\sim p\left(L\right)}\left[\log\left({}_{s}\left(L\right)\right)\right]\\ +{𝔼}_{H_{phantom}\sim p\left(H\right)}\left[\log\left(1-{}_{s}\left({}_{s}\left(H_{phantom}\right)\right)\right)\right] \end{array}$$

To encourage the output of the denoiser to match the clean phantom scans, we use an ℓ_1_ loss between the output and the ground truth image.


(2) $$\begin{array}{l} L_{1}\left(G_{d}\right)={𝔼}_{L,H\sim p\left(L,H\right)}\|H-{}_{d}\left(L\right){\|}_{1} \end{array}$$

Initializing the weights by pretraining can significantly ease the convergence of two interactive generators in both spatial and temporal space. However, the phantom scans still lack the essential features of scanning on a real patient.

#### 2.2.3. Learn Simulation Interacting With Denoiser: S2D ($H\to \hat{L}\to \hat{H}$)

We start with noise simulation to provide both noise and tissue features for training denoiser. We apply a discriminator *D*_*s*_ to train the simulator *G*_*s*_. We formulate the simulation objective as below.


(3) $$\begin{array}{l} L_{GAN}^{S2D}\left(G_{s},D_{s}\right)={𝔼}_{L\sim p\left(L\right)}\left[\log\left({}_{s}\left(L\right)\right)\right]\\ +{𝔼}_{H\sim p\left(H\right)}\left[\log\left(1-{}_{s}\left({}_{s}\left(H\right)\right)\right)\right] \end{array}$$

The simulator feeds its output into the denoiser during training. Thus, we formulate the denoising loss using a modified Equation 2 as below.


(4) $$\begin{array}{l} L_{1}^{S2D}\left(G_{d}\right)={𝔼}_{L,H\sim p\left(L,H\right)}\|H-{}_{d}\left({}_{s}\left(H\right)\right){\|}_{1} \end{array}$$

Besides the discriminator *D*_*s*_, we take advantage of the denoising performance as regularization feedback to indicate the quality of the simulation. As the simulation becomes better, the denoising is getting harder.

Furthermore, the simulator *D*_*s*_ in S2D takes the standard-dose scans from both phantom and patients as inputs. The phantom data applies a latent constrain to the *D*_*s*_ and stabilizes the training. Interacting with denoising encourages the simulator to generate realistic low-dose noise. Further, the denoise can benefit from taking the output of the simulator as additional training data, dynamically.

#### 2.2.4. Learn Denoising in Simulator: D2S ($L\to \hat{H}\to \hat{L}$)

The development of the training process from denoising to simulation has two significant varies from the cycle consistency study (6) (see Figure 2B). We first enable supervised learning to train the denoiser *G*_*d*_ using the standard-dose and the corresponding low-dose CT images. Compared to adversarial learning, supervised learning provides a stronger supervision signal to build an accurate denoiser. More importantly, the simulator in S2D produces the noise gradually close to the desired level during training. Thus, we can acquire various noise level images from the simulator, with which, the denoiser-self implicitly learns to restore clean CT scans for a range level of low-dose CT scans, rather than a specific noise level indicated in the training data. Therefore, the input to the denoiser *G*_*d*_ in D2S includes phantom low-dose and simulated patient low-dose images. We use a ℓ_1_ loss to train the denoiser *G*_*d*_. The ℓ_1_ loss encourages a pixel-wise match to the ground-truth. We illustrate the ℓ_1_ loss as below.


(5) $$\begin{array}{l} L_{1}^{D2S}\left(G_{d}\right)={𝔼}_{L,H\sim p\left(L,H\right)}\|H-{}_{d}\left(L\right){\|}_{1} \end{array}$$

Besides, we use adversarial learning to train the simulator in D2S to match the desired noise distribution in the actual low-dose CT scans. The objective to this adversarial learning the distribution is written as below.


(6) $$\begin{array}{l} L_{GAN}^{D2S}\left(G_{s},D_{s}\right)={𝔼}_{L\sim p\left(L\right)}\left[\log\left({}_{s}\left(L\right)\right)\right]\\ +{𝔼}_{Ĥ\sim p\left(H\right)}\left[\log\left(1-{}_{s}\left({}_{s}\left(Ĥ\right)\right)\right)\right] \end{array}$$

We develop the cyclic simulation and denoising training with regularizations in both directions and take advantage of both cycles $H\to \hat{L}\to \hat{H}$ and $L\to \hat{H}\to \hat{L}$. The total objective is illustrated below:


(7) $$\begin{array}{l} G_{s}^{*},G_{d}^{*}=arg\underset{G_{s},G_{d}}{\min}\underset{D_{s}}{\max}\lambda_{1}L_{GAN}^{S2D}\left(G_{s},D_{s}\right)+\lambda_{2}L_{GAN}^{D2S}\left(G_{s},D_{s}\right)\\ +\lambda_{3}L_{1}^{S2D}\left(G_{d}\right)+\lambda_{4}L_{1}^{D2S}\left(G_{d}\right) \end{array}$$

where λ indicates the weights of each loss. With these novel developments, the simulator and denoiser interact with each other in a cyclic self-learning manner to enable realistic noise simulation and accurate denoising for low-dose CT image.

## 3. Results

### 3.1. Datasets

We use *three* CT datasets during training and testing. The first dataset is obtained from the CT scanning on a single tissue-equivalent physical phantom model. This set contains various levels of low-dose series, scanned between 5 and 95 mAs with 5 mAs intervals. In this work, we simply use 20 mAs, 30 mAs, and 60 mAs low-dose phantoms for training noise simulation and evaluate the reality of various types of noise in Figure 1C. We also include the standard-dose (175 mAs) scans as the ground-truth. Each dose level of the phantom series produces 138 CT scans. The second dataset is a public Retrospective Image Registration Evaluation (RIRE) dataset. This dataset includes 388 standard-dose CT scans. We use 80% for training the simulator and denoiser in the proposed CSD and also task 20% for demonstrating the advantages of CSD over end-to-end training a denoiser in **Table 2**, where we simulate the low-dose noise by adding Gaussian noise on normal dose CT scans. We compute the corresponding standard variation of Gaussian noise for a specific mAs by following (7). Additionally, we acquire a real patient dataset including paired standard-dose (190 mAs) and low-dose (20 mAs) in a total of 432 CT scans. We use them for comparing various types of simulated noise in Figure 1C and evaluate the real noise removal performance of our approach in Table 1, where 250 scans are used for training and 182 scans are used for testing. Moreover, we randomly select 373 scans from this dataset combining with 20% of the RIRE dataset, in total 449 scans included to evaluate our CSD's generalizability in Table 2.

**Table 1:** The average real low-dose noise removal performance of the same deep neural network *trained* with Gaussian noise simulation and cyclic simulation and denoising (CSD) + physical phantom noise simulation, separately.

**PSNR (dB)/SSIM**		
**Noise level (mAs)**	**Trained with Gaussian**	**Trained with CSD** + **phantom**
30	24.47/0.7555	**26.10/0.8235**
60	23.03/0.6960	**25.51/0.7894**

*The best results are highlighted in bold*.

**Table 2:** The average Gaussian noise removal performance of the same deep neural network trained through the proposed CSD framework and the standard end-to-end manner, separately.

**PSNR (dB)/SSIM**		
**Noise level (mAs)**	**End-to-end training**	**CSD training**
30	31.93/0.9105	**32.05/0.9124**
60	33.33/0.9365	**33.92/0.9429**

*The best results are highlighted in bold*.

This dataset used de-identified data from a retrospective study which was HIPAA compliant and performed with University of Florida IRB approval as a minimal risk study with a waiver of informed consent.

### 3.2. Evaluation Metric

We develop CSD with U-net (8) for the simulator network *G*_*s*_ and DnCNN (9) for the denoiser network *G*_*d*_. We evaluate image denoising performance using Peak signal-to-noise ratio (PSNR) and image structural similarity index measure (SSIM).

### 3.3. Unsupervised Learning Performance on Real Low-Dose Noise Removal

Here, we aim to demonstrate that the proposed CSD framework in a combination with phantom can remove the real low-dose noise effectively. We first take the start-of-the-art medical image denoising network (9) as a baseline and train it with Gaussian simulated low-dose CT scans at different noise levels. Then, we build the *G*_*d*_ in CSD using the baseline's architecture and train it with paired low-dose and standard-dose phantom CT scans at the same noise levels as Gaussian simulation. We test each model on 182 real low-dose CT scans at the noise level of 20 mAs. The comparison results are shown in Figure 1C at 20 mAs and Table 1 at 30, 60 mAs noise levels. As one can see, the combination of the proposed CSD training framework and phantom simulation significantly outperforms the baseline with an average 1.56 dB improvement on PSNR across three different noise levels. Furthermore, as one can see in Figure 1C, the baseline network, which is trained with paired low-dose and standard-dose phantom scans, performs much worse than the model trained with both our CSD phantom and Gaussian simulation, which may be due to the lack of critical tissue features in the phantom scans. Notably, these results may indicate that *CSD, in combination with phantom simulation, can encourage the denoiser to learn both real low-dose noise features from phantom and tissue image features from patient scans*, simultaneously, and leading to real low-dose noise removal with greater accuracy and precision.

### 3.4. Evaluate CSD's Generalizability (Ablation Without *G*_*s*_)

**Figure 3:**
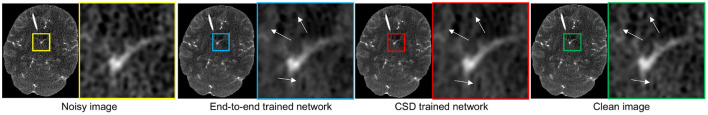
The visual comparison of the denoising performance between the network trained with end-to-end and the one trained with our CSD framework.

Here, we further evaluate the proposed CSD's generalizability to train a denoiser targeting the general simulated low-dose noise, such as Gaussian simulation. We still use the same baseline network to conduct this study. We use the standard end-to-end manner and our CSD framework to train two networks with the same architecture as the baseline, separately. Notably, to have a fair comparison, we only use original noisy CT scans in the training dataset as the input of the *G*_*d*_ in D2S cyclic training. Then, we compare the two networks to remove 30 and 60 mAs levels of Gaussian simulated low-dose noise from 449 CT scans. As one can see in Table 2, the model trained with our CSD can consistently outperform the one trained in an end-to-end manner, with an impressive average performance gain of 0.355 dB for PSNR. In addition, we also show a visual result comparison in Figure 3. As one can see, the denoiser *G*_*d*_ trained with our CSD framework can produce more realistic CT scans from its low-dose noisy version. This improvement can also be attributed to the interplay between the simulator and denoiser which *serve as regulators to each other* during the optimization process. These results suggest that starting with simulation may *create a live environment* from which the denoiser can learn high-validity representations to achieve a better denoising performance.

## 4. Conclusion

This paper proposed incorporating an anthropomorphic physical phantom model with generative deep learning networks for medical imaging, with a focus on realistic low-dose CT image restoration. A combination of an anthropomorphic physical model with deep generative adversarial networks can eliminate the needs of both actual low-dose patients and even other low-dose simulation CT scans to build an unsupervised learning framework for low-dose CT image restoration. More importantly, an anthropomorphic physical model CT scanning can abstract the unique noise properties of a particular CT imaging machine for the deep learning model to take CT machine domain variation into account during training. Eventually, with the interaction between a noise simulation network and a denoising network in cyclic training processing, the proposed deep learning model embraces realistic noise from low-dose phantom CT scans and tissue features from normal-dose patient CT scan into a single unified framework for building a state-of-the-art method for real low-dose CT image restoration.

## Data Availability Statement

The datasets presented in this article are not readily available because this study incorporates both private and public datasets. The anonymized private data are available from the corresponding authors upon reasonable request. The publicly available datasets presented in this study can be found in online repositories. The names of the repository/repositories and accession number(s) can be found in the article/Supplementary Material. Requests to access the datasets should be directed to RF, ruogu.fang@bme.ufl.edu.

## Ethics Statement

This human subject retrospective study was HIPAA compliant and performed with University of Florida IRB approval as a minimal risk study with a waiver of informed consent.

## Author Contributions

PL, YX, and RF contributed to conception and design of the study. PL, GF, YX, and J-BN organized the database. PL, LX, and ZL contributed to the software used in this study. PL performed the statistical analysis. IB and CO provided digital scanning data of the anthropomorphic physical phantom. PL and GF wrote the first draft of the manuscript. PL, GF, YX, and RF wrote sections of the manuscript. RF was responsible for the supervision, project administration, and funding acquisition. All authors contributed to manuscript revision, read, and approved the submitted version.

## Funding

This material is based upon work supported by the National Science Foundation under grant no. NSF 1908299.

## Conflict of Interest

The authors declare that the research was conducted in the absence of any commercial or financial relationships that could be construed as a potential conflict of interest.

## Publisher's Note

All claims expressed in this article are solely those of the authors and do not necessarily represent those of their affiliated organizations, or those of the publisher, the editors and the reviewers. Any product that may be evaluated in this article, or claim that may be made by its manufacturer, is not guaranteed or endorsed by the publisher.
