# Supplementary material for: PIMA-CT: Physical Model-Aware Cyclic Simulation and Denoising for Ultra-Low-Dose CT Restoration
Source: Front Radiol. 2022 May 25;2:904601. doi: 10.3389/fradi.2022.904601 (PMC10365089; doi:10.3389/fradi.2022.904601)
Supplement: Supplementary file 1 [file Data_Sheet_1.PDF]

PIMA-CT: Physical Model-Aware Cyclic Simulation and Denoising for Ultra-low-dose CT Restoration: Supplementary Material

1 NETWORK ARCHITECTURE AND TRAINING DETAILS

For the simulator G_s, we adopt a standard u-shape architecture network (e.g., U-net) to map images from a denoised domain to a noisy domain. U-shape structure can combine the low-level with high-level features to generate realistic images. For the denoiser G_d, we use a stacked CNN (e.g., DnCNN) to restore noisy-free images by learning both noise and brain features from noisy inputs. A simple stacked convolutional network allows us to focus on the development of the insight of the proposed CSD framework.

For discriminators D_s, we use a patch-based discriminator to classify (NxN) image patches as either simulated or real. We apply learning rate 0.0001 and use 64×64 size of image patches as the input to train networks. We choose Adam as the optimizer and 128 as the batch size for all networks training. For loss weights λ, we experimentally apply 1, 1, 20, and 1 for $\mathcal{L}_{GAN}^{S2D}$, $\mathcal{L}_1^{S2D}$, $\mathcal{L}_1^{D2S}$, and $\mathcal{L}_{GAN}^{D2S}$, separately. We use these weights to balance the simulation and denoising. We weight the simulation more in S2D while applying more weights for denoising in D2S.

We train our CSD with 3 iterations and 50 epochs each. In the first iteration, we only use the noisy phantom as the input of the G_d in the D2S cycle for training. This strategy allows G_d to intensively learn removing the complicated real noise from CT scans. In the following iterations, we combine the noisy patient scans generated by G_s to continue the training of G_d in D2S. This strategy enables G_d to learn both noise and brain features, simultaneously, leading to a superior denoising performance.

2 VARIOUS DOSE LEVELS OF PHANTOM SCANS

In this work, we use phantom scans to obtain realistic low-dose noise. Phantom scans allow us to step further for the understanding of the real noise properties. Thus, we demonstrate the intensity changes of the various levels of phantom scans in Figure S1. As one can see, when using the higher level of dose, the less noise is presented on the scans. The 175 mAs is considered as a normal dose level. Besides, the phantom scans lack the tissue features in constant to the scans on patients. The defect prevents the development of a denoising model using phantom scans directly, which motivated us to conduct this study.

3 SAMPLES OF REAL PAIRED LOW- AND STANDARD-DOSE PATIENT SCANS

We apply low and standard radiation dose CT scanning on patients to obtain the real paired low- and standard-dose patient CT scans. We show more samples from this dataset in Figure S2. By comparing with phantom scans, as one can see, the real low-dose CT scans have not only noise but also clear tissue and blood-vessel features. Therefore, to ideally restore high-quality images from low-dose CT scans, the model has to be able to learn both noise and tissue features, simultaneously. This intuition encourages us to develop a novel framework to embrace the noise and tissue features together for training a denoising model.

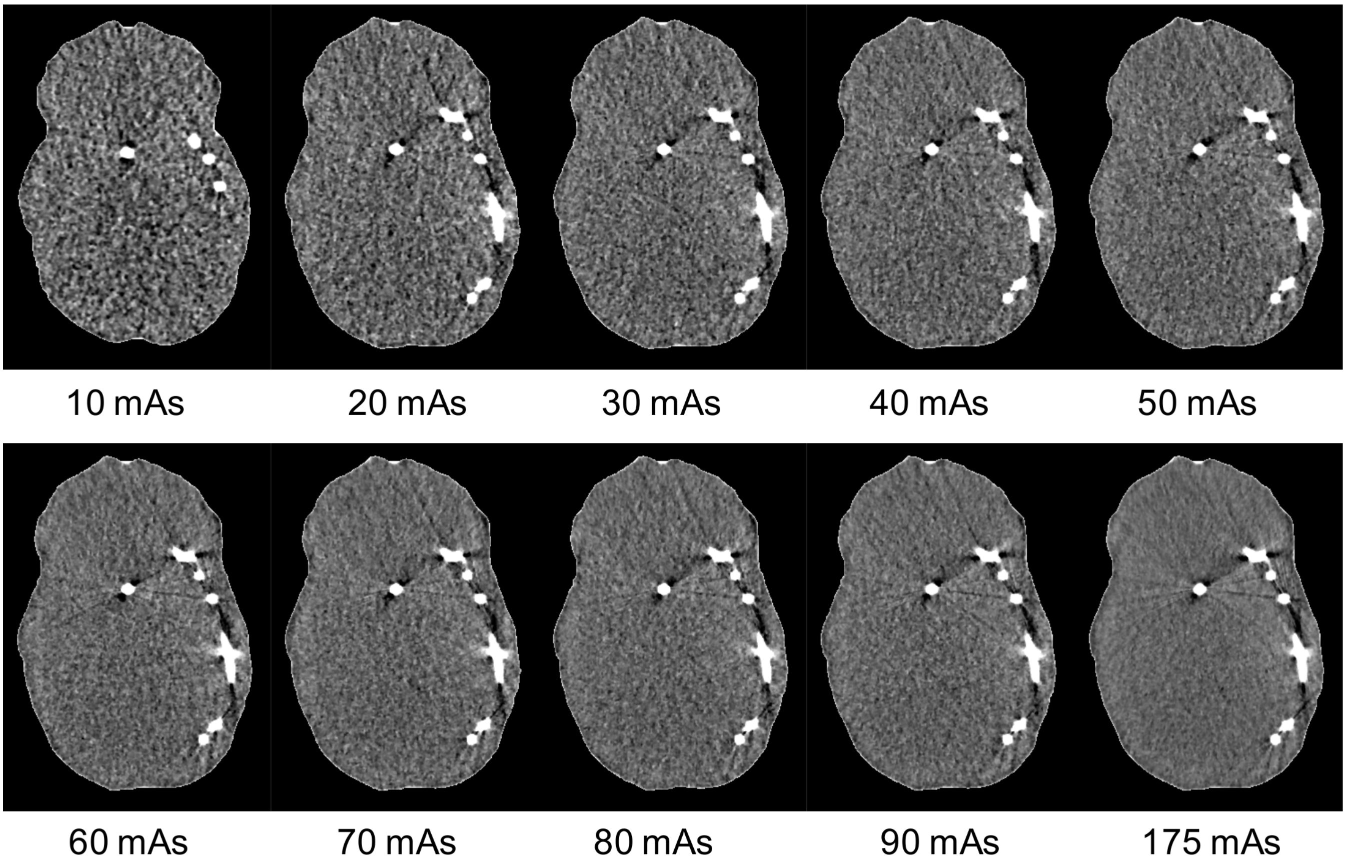

Figure S1. The visual comparison of various dose levels of a phantom CT scan from 10 mAs to 175 mAs.

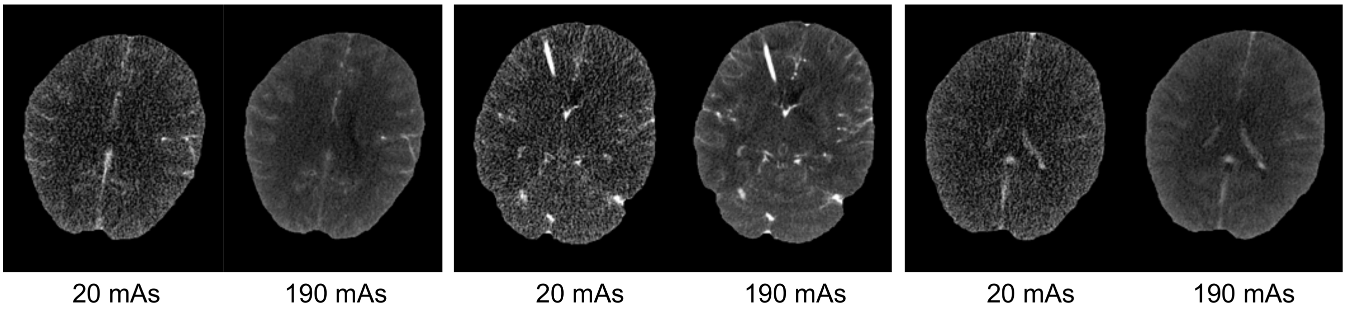

Figure S2. The visual comparison of different paired real low-dose and standard-dose patient CT scanning slices from one subject.

4 VISUAL RESULTS OF LOW-DOSE SIMULATION

In this study, we mainly aim to restore high-quality images from low-dose CT scans. We take advantage of noise simulation to assist image restoration. Particularly, we weight more denoising than the simulation. We demonstrate our simulation results in Figure S3, which indicates the effectiveness of simulation that can transfer the noise from low-dose phantom to standard-dose patient CT scans. In future work, we can shift the weights to generate more realistic simulations.

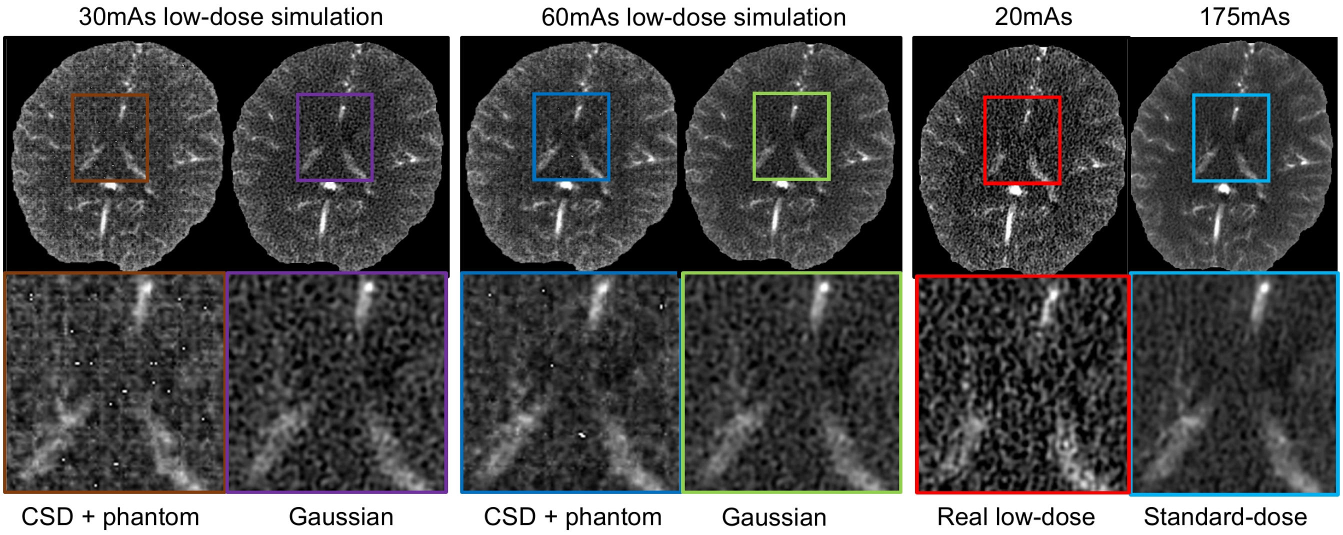

Figure S3. The visual comparison of the low-dose simulations using our CSD framework combining with phantom and Gaussian simulation.

5 VISUAL RESULTS OF LOW-DOSE CT IMAGE DENOISING

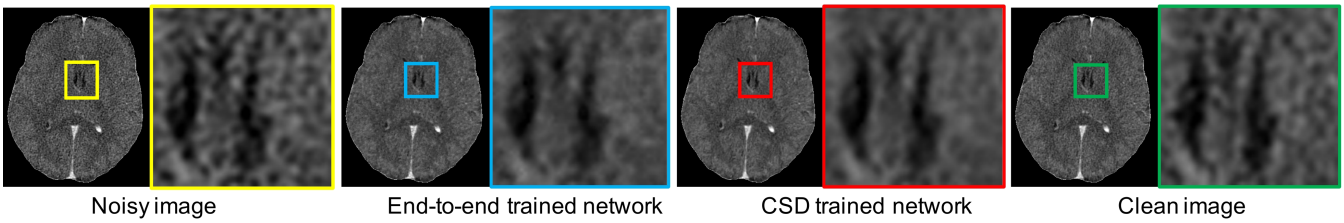

Figure S4. The visual comparison of the denoising performance on a Gaussian simulated low-dose (60 mAs) CT image between the network trained with end-to-end and the one trained with our CSD framework.

The proposed CSD framework provides an interactive model training environment. This environment plays like a "classroom" in which noise and tissue features are learning material, and the interactive simulator and denoiser are a teacher and a student. The interactive learning strategy facilities a profound understanding of the material. Thus, the denoiser trained through the CSD can not only remove the noise effectively but also restore the fine tissue features accurately (see Figure S4).
